# *Cellulophaga algicola* alginate lyase and *Pseudomonas aeruginosa* Psl glycoside hydrolase inhibit biofilm formation by *Pseudomonas aeruginosa* CF2843 on three-dimensional aggregates of lung epithelial cells

**DOI:** 10.1016/j.bioflm.2025.100265

**Published:** 2025-02-22

**Authors:** Neetu, Shilpee Pal, Srikrishna Subramanian, T.N.C. Ramya

**Affiliations:** a CSIR- Institute of Microbial Technology, Sector 39-A, Chandigarh, 160036, India; b Academy of Scientific & Innovative Research (AcSIR), Ghaziabad, Uttar Pradesh, 201002, India

**Keywords:** Cystic fibrosis, *Pseudomonas aeruginosa*, Alginate lyase, Psl hydrolase, 3D model system, RCCS

## Abstract

*Pseudomonas aeruginosa* is an opportunistic pathogen that produces a biofilm containing the polysaccharides, alginate, Psl, and Pel, and causes chronic lung infection in cystic fibrosis patients. Others and we have previously explored the use of alginate lyases in inhibiting *P. aeruginosa* biofilm formation on plastic and lung epithelial cell monolayers. We now employ a more physiologically representative model system, i.e., three-dimensional aggregates of A549 lung epithelial cells cultured under conditions of microgravity in a rotary cell culture system to mimic the natural lung environment, and a previously isolated clinical strain, *Pseudomonas aeruginosa* CF2843 that we engineered by transposon-mediated integration to express Green Fluorescent Protein and for which we also report the complete genome sequence. Immunostaining and lectin binding studies indicated that the three-dimensional cell aggregates harbored sialylated and fucosylated epitopes as well as Muc1, Muc5Ac, and β-catenin on their surfaces, suggestive of mucin secretion and the presence of tight junctions, hallmark features of lung epithelial tissue. Using this validated model system with confocal microscopy and viable bacterial counts as readouts, we demonstrated that *Cellulophaga algicola* alginate lyase and *Pseudomonas aeruginosa* Psl glycoside hydrolase, but not *Pseudomonas aeruginosa* Pel glycoside hydrolase, inhibit biofilm formation by *Pseudomonas aeruginosa* on three-dimensional lung epithelial cell aggregates.

## Highlights

• We obtained 3D aggregates of A549 cells by culturing under microgravity conditions.
• We obtained GFP expressing *P. aeruginosa* CF2843 by transposon-mediated integration.
• We assessed how CaAly, PslG, and PelAh affect biofilm formation by *P. aeruginosa*.
• CaAly and PslG inhibit biofilm formation by *P. aeruginosa* CF2843 on 3D lung culture.
• PelAh only displayed a modest effect on biofilm formation by *P. aeruginosa* CF2843.

## 1 Introduction

Cystic fibrosis is a monogenic autosomal recessive disease caused by a mutation in the Cystic Fibrosis Transmembrane Conductance Regulator gene (*CFTR*), and it is the most common multiorgan hereditary disorder worldwide [1,2]. The disease leads to the deposition of a thick mucus layer in the lungs and many other organs, which generates a conducive environment for opportunistic bacteria, and results in tissue damage and loss of organ function [3]. Common opportunistic bacteria in cystic fibrosis patients are *Pseudomonas aeruginosa* (found in 80% of cystic fibrosis patients), *Staphylococcus aureus,* and *Haemophilus influenzae* [4]. *P. aeruginosa* grows as a mucoid form in the cystic fibrosis lung and forms a biofilm, a group of bacteria embedded in a self-produced matrix of polysaccharides, proteins, and DNA, which is highly resistant to antibiotics and to many innate and adaptive host immune factors [5,6].

The polysaccharides in the *P. aeruginosa* biofilm include alginate, Psl, and Pel. Alginate is one of the main polysaccharides produced by the mucoid form of *P. aeruginosa* as a consequence of a mutation in the *mucA* gene of the alginate biosynthesis regulation operon [7]. Alginate is a negatively charged polymer made up of β-d-mannuronic acid and α-l-guluronic acid, whose ratio affects the viscoelastic properties of the biofilm [8]. Alginate production helps in the maturation of the biofilm, provides protection from opsonization and phagocytosis, and acts as a barrier against antibiotics [9,10]. Psl is a neutral polysaccharide comprising d-mannose, d-glucose, and d-rhamnose [11] and is involved in early biofilm development [12]. Pel is a cationic polysaccharide comprising d-glucosamine and *N-*acetyl-d-galactosamine [13]. Polysaccharides, Psl and Pel, are essential for cell-cell interaction, surface attachment, and biofilm integrity; they are present in the biofilm periphery of microcolonies [14,15] and offer structural redundancy within the biofilm matrix in both mucoid and non-mucoid *P. aeruginosa* [14,[16], [17], [18]].

Antimicrobial and enzymatic approaches targeting the biofilm matrix can be promising alternatives to traditional antibiotics [19]. Alginate lyases, a class of polysaccharide lyases that cleaves alginate by β-elimination into oligosaccharides containing 4-deoxy-L-*erythro*-hex-4-enopyranosyluronic acid at the non-reducing end [20] have been explored for their ability, in combination with DNase or clinically relevant antibiotics, to disrupt the biofilm matrix formed by *P. aeruginosa* in cystic fibrosis [[21], [22], [23]]. PslG, encoded by a gene of the *psl* operon, is a periplasmic protein with a glycoside hydrolase domain and a carbohydrate-binding module, which likely degrades Psl by hydrolyzing the glycosidic bond between β-D-Man and α-L-Rha, and causes biofilm disassembly in *P. aeruginosa* [24,25]. PelA, encoded by a gene of the *pel* operon, is a protein with a deacetylase domain and an endo-α-1,4-N-acetylgalactosaminidase domain, which inhibits biofilm formation, decreases biofilm mass, and disrupts preformed biofilms in *P. aeruginosa* [[26], [27], [28], [29]].

Although several studies have explored the potential of these enzymes in inhibiting biofilm formation by *P. aeruginosa* on plastic surfaces, there is a dearth of studies exploring the potential of these enzymes in a model system more closely mimicking the milieu in the cystic fibrosis lung. Previously, we demonstrated the potential of *Ca*Aly (*Cellulophaga algicola* alginate lyase) in inhibiting *P. aeruginosa* biofilm formation on A549 lung epithelial cells (A549) [30]. However, our experimental set-up comprised A549 cells cultured as two-dimensional monolayers. Cells in monolayers are known to lose their key phenotypic characteristics that mediate interactions with the bacteria in a biofilm [31,32], and thus, such a model might not sufficiently mimic the *in vivo* lung tissue architecture or reflect *in vivo* responses.

Three-dimensional (3D) cell cultures with a tissue-like architecture and/or tissue-like micro-environment are an alternative solution for studying host-pathogen interactions while overcoming the challenges inherent to using conventional monolayers, and previously, a low-shear stress 3D model of A549 lung epithelial cells cultured under conditions of microgravity using a rotating wall vessel and dextran microcarrier beads coated with collagen was reported to mimic the *in vivo* microenvironment in terms of phenotypic characteristics, apical-basolateral polarity, and secretion of mucin (unlike “flat surface” grown cells in monolayers) [[33], [34], [35], [36], [37], [38]]. We have employed the same 3D lung epithelial cell culture system together with a Green Fluorescent Protein (GFP)-expressing clinical strain *P. aeruginosa* CF2843 to assess the inhibitory activity of the carbohydrate-active enzymes, *Ca*Aly, PslG, and PelAh (PelA endo-α-1,4-N-acetylgalactosaminidase domain), on *P. aeruginosa* biofilm formation. We report the complete genome sequence of the GFP-integrated *P. aeruginosa* CF2843 and demonstrate that *Ca*Aly and PslG but not PelAh inhibit biofilm formation by *P. aeruginosa* CF2843 on the 3D aggregates of A549 lung epithelial cells.

## 2 Results

### 2.1 Transposon-mediated integration of *gfp* gene in a clinical *P. aeruginosa* strain from cystic fibrosis sputum

**Fig. 1:**
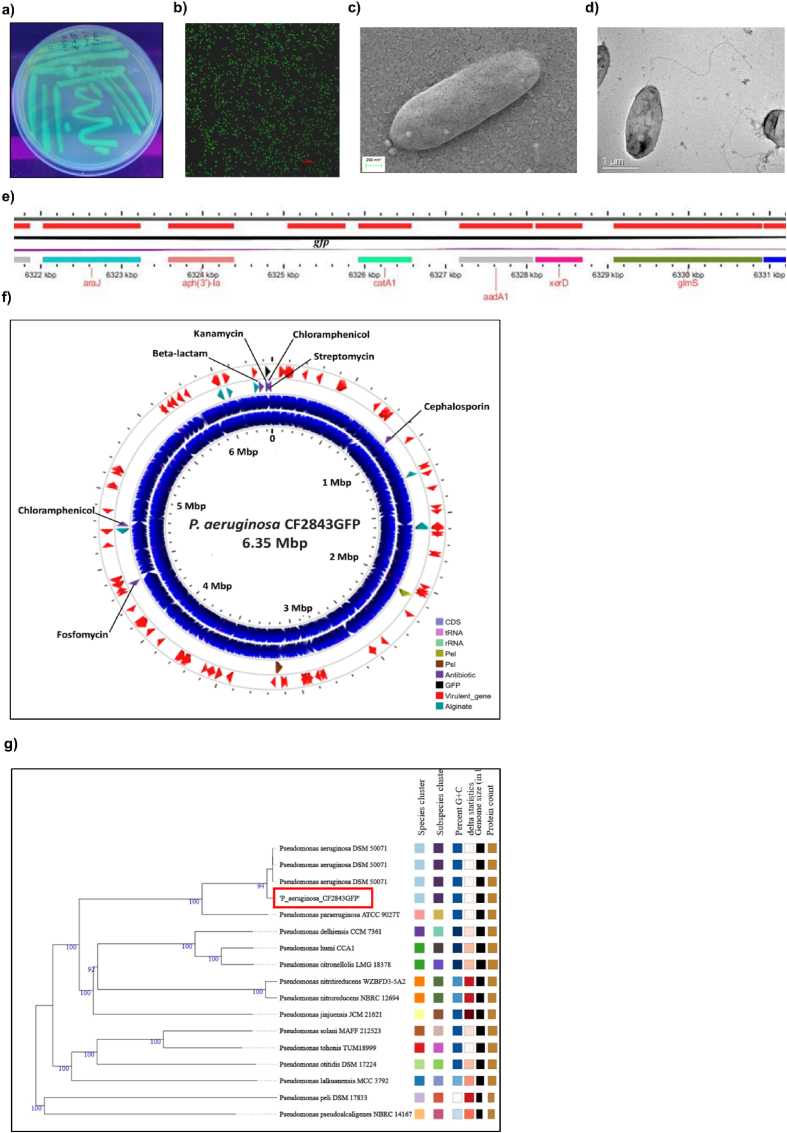
**Integration of *gfp* gene in the clinical strain *P. aeruginosa* CF2843: a)** LB agar plate with *P. aeruginosa* CF2843GFP under UV light **b)** Confocal microscopy image indicating the fluorescence of *P. aeruginosa* CF2843GFP bacteria using a laser of 480 nm wavelength (scale bar: 20 μm) **c)** Scanning Electron Microscopy image of *P. aeruginosa* CF2843GFP (scale bar: 200 nm) **d)** Transmission Electron Microscopy image of *P. aeruginosa* CF2843GFP (scale bar: 1 μm) **e)** Zoomed view of the *P. aeruginosa* CF2843GFP genome indicating the region where the *gfp* gene has been integrated **f)** Circular map view of the genome of *P. aeruginosa* CF2843GFP showing the CDS, tRNA, rRNA, Psl biosynthesis pathway genes, Pel biosynthesis pathway genes, alginate biosynthesis pathway genes, antimicrobial resistance genes, virulence genes, and *gfp*. **g)** Phylogenetic positioning of *P. aeruginosa* CF2843GFP as per the TYGS server [40].

We previously reported the clinical strain *P. aeruginosa* CF2843, a sputum isolate from a cystic fibrosis patient [39]. We used this strain in this study and successfully integrated the *gfp* (green fluorescent protein) gene into this bacterial genome using transposon-mediated integration to facilitate ease of imaging. We isolated *P. aeruginosa* CF2843GFP by screening colonies for GFP fluorescence following antibiotic selection (Fig. 1a and b). The intensity of GFP fluorescence was constant upon subculturing five times in *P. aeruginosa* CF2843GFP cultured with or without kanamycin, indicating stable genome integration (Fig. S1a). SEM and TEM analysis showed flagellated rods, as expected, with cell lengths of 1.77 ± 0.052 mm and 1.57 ± 0.34 mm (p-value of 0.052 in a two-sample equal variance *t*-test, n = 40) for *P. aeruginosa* CF2843 and *P. aeruginosa* CF2843GFP, respectively, and no other phenotypic change was noted upon *gfp* integration (Fig. 1c and d and Fig. S1b, S1c, Table S1). We also confirmed *gfp* integration in *P. aeruginosa* CF2843GFP by genome sequencing, assembly, and annotation. As expected, *gfp* was inserted in the *att*Tn7 site adjacent to the coding region of the *glmS* gene (Fig. 1e).

### 2.2 Genomic features of *P. aeruginosa* CF2843GFP and comparative genomics with other *P. aeruginosa* genomes

The sequenced reads of *P. aeruginosa* CF2843GFP assembled into a single complete circular genome of 6,350,848 bp with a GC content of 66.46% and functional annotation using Bakta V1.5.1 revealed a coding density of 90.3% and 5792 coding sequences (CDS) (Fig. 1f). TYGS analysis [40] indicated that CF2843GFP is most closely related to *P. aeruginosa* DSM 50071 (Fig. 1g). A phylogenetic tree based on 6 MLST genes of 144 *P. aeruginosa* genomes (73 from non-CF humans and 71 from cystic fibrosis patients) indicated that *P. aeruginosa* CF2843GFP is most closely related to *P. aeruginosa* strain PALA56-22839, which was isolated from a cystic fibrosis patient, and similar results were obtained with an SNP tree of these genomes (Figs. S2a and S2b). These phylogenetic trees, however, did not exhibit segregated clustering of *P. aeruginosa* strains from non-CF individuals and cystic fibrosis patients, indicating the absence of significant adaptations at the genome level to the cystic fibrosis lung environment.

Analysis of the antimicrobial resistance (AMR) genes by RGI, ARGfam, and Amrfinder and combination result indicated that the *P. aeruginosa* CF2843GFP genome contains genes that confer resistance to Beta-lactam, Aminoglycoside, Fosfomycin, and Phenicol antibiotics (Fig. S3a). Antimicrobial susceptibility testing of *P. aeruginosa* CF2843GFP confirmed resistance to a few of these antibiotics - Beta-lactam, Aminoglycoside, and Nitrofuran (Fig. S3b, S3c-e). A comparative analysis of the AMR genes in the 144 aforementioned *P. aeruginosa* genomes indicated a greater prevalence of AMR genes in isolates from cystic fibrosis patients than in non-CF individuals (Tables S2–6).

Functional annotation and analysis of the *P. aeruginosa* CF2843GFP genome also indicated the presence of alginate, Psl, and Pel biosynthetic pathways; alginate and Pel biosynthetic pathways were complete, but three genes with unknown functions, *pslJ*, *pslK*, and *pslL*, were absent in the Psl biosynthetic pathway (Figs. S4a–c). A comparative analysis of these genes in the 144 aforementioned *P. aeruginosa* genomes again indicated a higher representation of complete gene pathways as well as of individual genes of these pathways in the genomes of strains isolated from cystic fibrosis patients as compared to those from non-CF individuals (Tables S7–12).

### 2.3 Comparison of the phenotypic surface characteristics of A549 monolayers and 3D cell aggregates

**Fig. 2:**
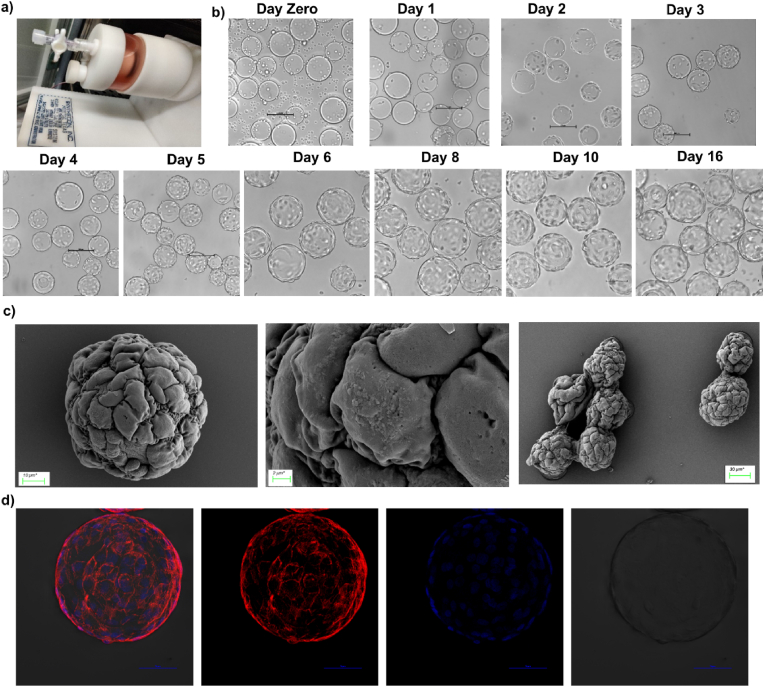
**Culturing the 3D aggregates of A549 lung epithelial cell: a)** The 55 ml Slow-Turning Lateral Vessel (STLV) of the Rotary Cell Culture System (Synthecon) **b)** Phase contrast microscopy image of microcarrier beads with A549 cells in the vessel from day zero to day 16 (Scale bar: 100 μm) **c)** Scanning Electron Microscopy image of 3D aggregates of A549 cells on day 13 at different scales of magnification (scale bar: 10 μm, 2 μm, and 30 μm, respectively). **d)** Confocal microscopy images of 3D aggregates of A549 stained with AF568-labelled phalloidin (red) and DAPI (blue). Scale bar: 100 μm. (For interpretation of the references to colour in this figure legend, the reader is referred to the Web version of this article.)

We cultured A549 monolayers on standard TC-treated plastic and A549 3D cell aggregates in an RCCS system using collagen-coated dextran microcarrier beads as a scaffold, as previously reported [33]. Cells adhered to the microcarrier beads within 24 hours of culturing in the RCCS system; microcarrier beads were fully covered with healthy 3D cell aggregates on days 9–16 (Fig. 2a and b). We visualized the A549 3D cell aggregates by scanning electron microscopy, which suggested the presence of a single layer of cells with secretory vesicles, intercellular and inter-aggregate contact, and architectural complexity (Fig. 2c), as earlier reported [33]. We also visualized the 3D aggregates on the beads by staining the F-actin (Alexa Flour 568 Phalloidin: red) and the nuclei (DAPI: blue) in the cells (Fig. 2d).

**Fig. 3:**
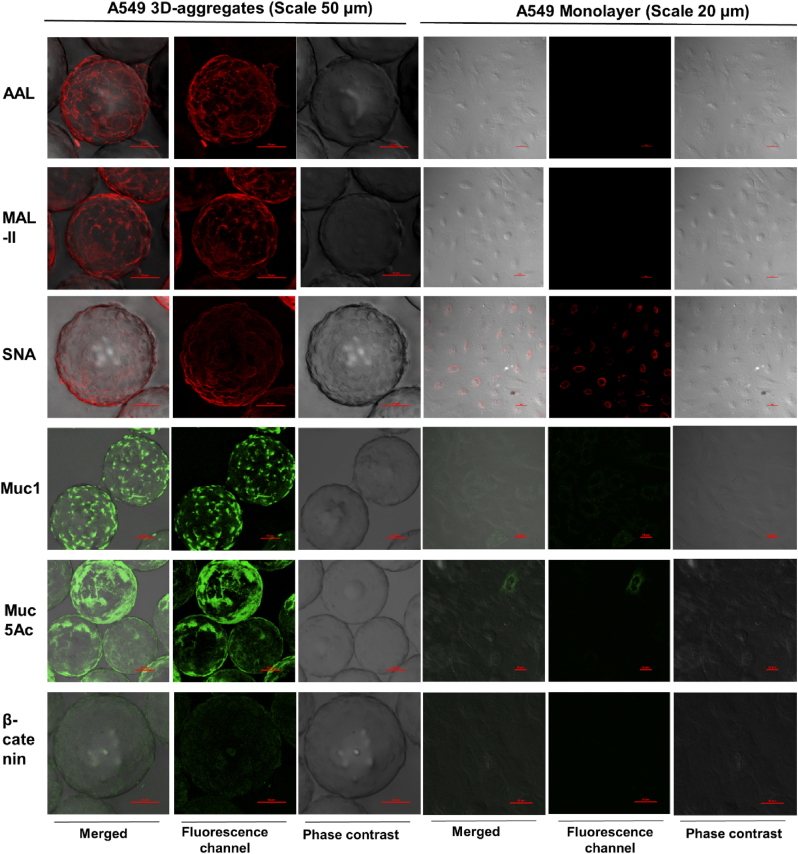
**Characterization of 3D aggregates of A549 cells.** Confocal microscopy images of 3D aggregates and monolayers of A549 cells stained with AAL (red), MAL-II (red), and SNA (red), anti-Muc1 (green), anti-Muc5AC (green), and anti-β-catenin (green). Scale bars for 3D and monolayers are 50 μm and 20 μm, respectively. (For interpretation of the references to colour in this figure legend, the reader is referred to the Web version of this article.)

We compared the cell surface glycosylation on A549 monolayers and 3D cell aggregates and found that 3D cell aggregates showed more cell surface fucosylation (as detected with *Aleuria aurantia* lectin) and sialylation (as detected with *Sambucus nigra* agglutinin and *Maackia amurensis* agglutinin, which recognize α2,6-sialic acids and α2,3-sialic acids, respectively) as compared to A549 monolayers (Fig. 3). A549 3D cell aggregates also displayed more tight junctions and cell surface mucin than A549 monolayers upon immunostaining with anti-beta-catenin and anti-Muc1/anti-Muc5Ac (Fig. 3), as expected [33,35].

### 2.4 Effect of *Ca*Aly, PslG, and PelAh on *P. aeruginosa* CF2843GFP biofilm formation on A549 monolayers and 3D cell aggregates

**Fig. 4:**
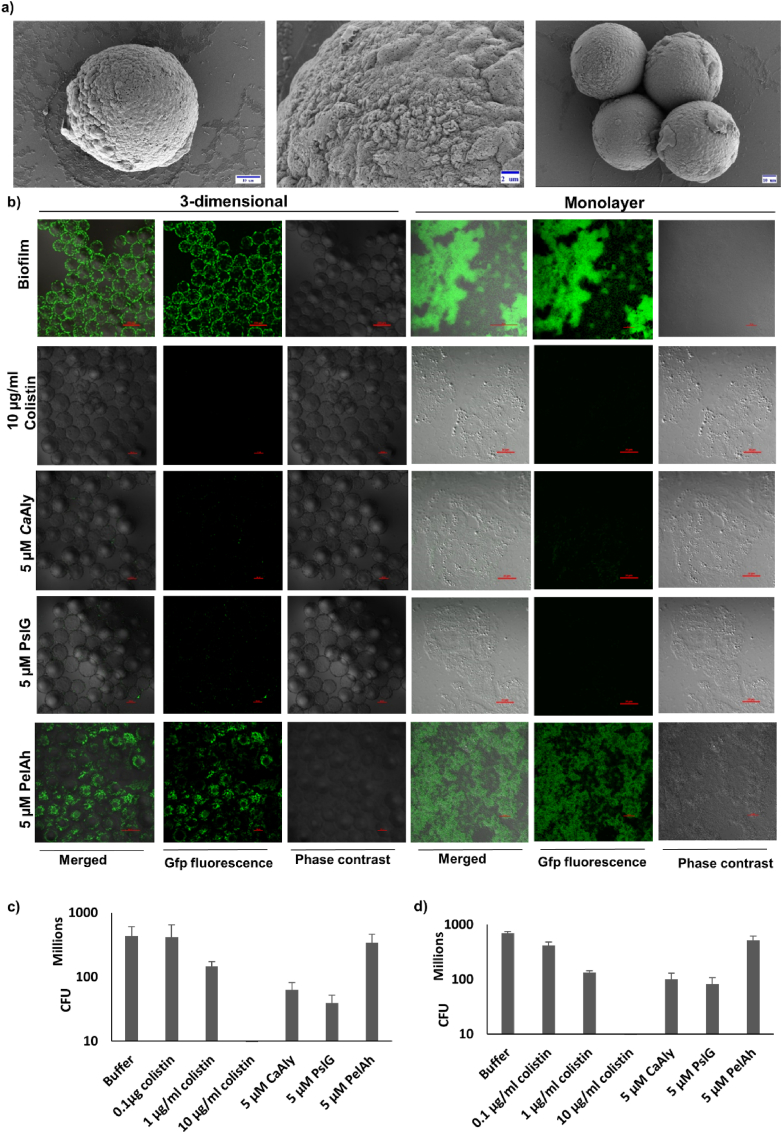
**Biofilm formation *by P. aeruginosa* CF2843GFP on A549 lung epithelial cells and its inhibition. a)** Scanning Electron Microscopy image of 3D aggregates of A549 cells (harvested on day 13) covered with *P. aeruginosa* CF2843GFP biofilm-like structures at different scales of magnification. Scale bars: 10 μm, 2 μm, and 10 μm. **b)** Confocal microscopy image showing *P. aeruginosa* CF2843GFP biofilms on 3D aggregates and monolayers of A549 cells after 12 hours of incubation with TBS (Scale bars for 3D aggregates and monolayers are 200 μm and 20 μm, respectively), 10 μg/ml colistin (Scale bars for 3D aggregates and monolayers are 100 μm and 20 μm, respectively), 5 μM *Ca*Aly (Scale bars for 3D aggregates and monolayers are 100 μm and 20 μm, respectively), 5 μM PslG (Scale bars for 3D aggregates and monolayers are 100 μm and 20 μm, respectively), 5 μM PelAh (Scale bars for 3D aggregates and monolayers are 200 μm and 20 μm, respectively). **c)** Viable bacterial counts of *P. aeruginosa* obtained following incubation of 3D aggregates of A549 lung epithelial cells with *P. aeruginosa* CF2843 and TBS, 0.1 μg/ml colistin, 1 μg/ml colistin, and 10 μg/ml colistin, 5 μM *Ca*Aly, 5 μM PslG, and 5 μM PelAh. **d)** Viable bacterial counts of *P. aeruginosa* obtained following incubation of monolayers of A549 lung epithelial cells with *P. aeruginosa* CF2843 and TBS, 0.1 μg/ml colistin, 1 μg/ml colistin, and 10 μg/ml colistin, 5 μM *Ca*Aly, 5 μM PslG, and 5 μM PelAh.

*P. aeruginosa* CF2843GFP formed biofilm-like structures on 3D cell aggregates, similar to *P. aeruginosa* PAO1 [33], and we visualized these using SEM (Fig. 4a). We also used confocal microscopy to visualize biofilm formation by *P. aeruginosa* CF2843GFP on A549 monolayers and 3D cell aggregates (Fig. 4b). The addition of 10 μg/ml of the commercially available antibiotic colistin completely abrogated GFP fluorescence, indicating the absence of A549-associated *P. aeruginosa* CF2843GFP, whereas lower concentrations of colistin (0.1 μg/ml and 1 μg/ml) resulted in slight to moderate depletion of the GFP fluorescence associated with the A549 cells (Fig. 4b, Fig S5a, Fig S5b). Importantly, treatment of A549 cells with 5 μM *Ca*Aly or PslG also resulted in complete abrogation of GFP fluorescence, indicating the absence of A549-associated *P. aeruginosa* CF2843GFP presumably due to inhibition of biofilm formation (Fig. 4b, Fig. S5c). Similar results were obtained with both A549 monolayers and A549 3D aggregates (Fig. 4b). We observed no change in the GFP fluorescence upon incubation of A549 monolayers or 3D aggregates with 5 μM PelAh, suggesting that PelAh did not similarly inhibit biofilm formation by *P. aeruginosa* CF2843GFP (Fig. 4b, Fig. S5c).

We also used viable bacterial counts to get a quantitative measure of the effect of *Ca*Aly, PslG, and PelAh on biofilm formation on A549 monolayers and 3D cell aggregates by *P. aeruginosa* CF2843 (wild-type clinical strain without any genome modification). We measured the viable cell counts of *P. aeruginosa* CF2843 bacteria associated with biofilms on the cells after washing away the planktonic bacteria and noted a complete reduction in the viable bacterial counts associated with both A549 monolayers and A549 3D cell aggregates when treated with 10 μg/ml colistin, and a significant reduction of 80–90% (two-tailed paired *t*-test with p-value<0.05) in the viable bacterial counts associated with both A549 monolayers and A549 3D cell aggregates when treated with 5 μM *Ca*Aly, or 5 μM PslG (Fig. 4c and d). No significant inhibition was found in A549 monolayers and 3D cell aggregates upon treatment with 5 μM PelAh (two-tailed paired *t*-test with p-value< 0.05). We also noted a significant but modest reduction of 40–50% (two-tailed paired *t*-test with p-value< 0.05) in the viable cell count upon treatment with 1 μg/ml colistin in A549 monolayers and 3D aggregates (Fig. 4c and d).

## 3 Discussion

*Pseudomonas aeruginosa* is an opportunistic pathogen that infects various body parts, particularly the respiratory tract [41,42]. Chronic *P. aeruginosa* infections are a scourge in cystic fibrosis, and there is a high prevalence of infections by biofilm-producing strains of *P. aeruginosa* in immunocompromised hospitalized patients [[43], [44], [45]]. The ability of *P. aeruginosa* to produce biofilms enables chronic infections in cystic fibrosis and is also the primary reason for the low efficacy of antibiotics in clearing these infections [46,47]. Carbohydrate-active enzymes that act on *P. aeruginosa* biofilm components are, therefore, obvious therapeutic options to be explored.

Genes of the alginate, Psl, and Pel biosynthesis pathways play a significant role in biofilm formation; the genes *algD*, *algU*, *algL*, *pslA*, *pslD*, *pelA*, and *pelF* have been reported to be significantly prevalent in clinical isolates [46,47]. Further, alginate is known to be the predominant biofilm component in mucoid *P. aeruginosa* [48]. Psl has also been reported to be a critical component of the biofilm matrix in these strains [17]. The genome sequence of *P. aeruginosa* CF2843 indicates that it is non-mucoid (no mutation in *mucA* that might lead to the expression of a truncated MucA protein), albeit it has a mucus-like colony morphology on agar, suggestive of high expression of alginate. Whereas *Ca*Aly and PslG robustly inhibited biofilm formation, *P. aeruginosa* Pel glycoside hydrolase (PelAh) did not do so in our study. Possible explanations might include sub-optimal activity of the recombinant PelAh (we did not assay the functional activity of the PelAh before use, and so cannot exclude the possibility that PelAh was not functionally active) and/or a lower proportion of the Pel polysaccharide in *P. aeruginosa* CF2843 (attempts to quantify alginate, Psl, and Pel by staining them with CBM16 alginate-binding domain from *Wenyingzhuangia fucanilytica* HPA (*Hippeastrum hybrid* agglutinin), and WFA (*Wisteria floroibunda* agglutinin) were not successful, and gene expression from alginate, Psl, and Pel biosynthesis gene clusters was not assessed by RT-PCR or RNA sequencing). Albeit all genes in the Pel biosynthesis pathway were found to be present in our isolate *P. aeruginosa* CF2843 and all genes of the Pel biosynthesis pathway are essential for *P. aeruginosa* to make Pel [49,50], it is possible that our clinical isolate harbors mutations in the genes required for Pel synthesis or that Pel synthesis is negatively regulated.

Several previous studies have assessed the inhibitory effect of alginate lyases, PslG, and PelAh alone or in combination with antibiotics on biofilms formed *in vitro* on plastic surfaces or in *P. aeruginosa* wound infections in mice [51], and we have also previously demonstrated the biofilm inhibitory effect of *Ca*Aly and PslG on A549 monolayers [39]. PslG and PelAh inhibit biofilm formation and disassemble pre-formed biofilms of a wide range of *Pseudomonas* sp. *in vitro* at nanomolar concentrations, sensitize the biofilms to antibiotics, increase neutrophil killing without any toxicity to human cells, and reduce the bacterial burden when administered together with antibiotic in mice [24,25,[52], [53], [54]]. Studies have also shown the anti-biofilm effect of alginate lyases alone or in combination with antibiotics or anti-microbial peptides [[21], [22], [23],^30^,[55], [56], [57], [58], [59], [60]] albeit a previous study indicated that the biofilm dispersion by alginate lyase might be catalysis-independent [61]. Encouragingly, an attenuated and genetically engineered *Mycoplasma pneumoniae* expressing recombinant PslG, PelAh, A1-II alginate lyase, and a bacteriocin was also recently efficacious in a *P. aeruginosa* infected mouse model [62]. There, remains, however, a dearth of studies on cystic fibrosis models. In this study, we have demonstrated the ability of *C. algicola* alginate lyase (*Ca*Aly) and *P. aeruginosa* Psl glycoside hydrolase (PslG) to inhibit the biofilm formed by the cystic fibrosis isolate *P. aeruginosa* CF2843 in a 3D cell culture model of lung epithelial cells. In summary, *Ca*Aly and PslG similarly inhibit biofilm formation by *P. aeruginosa* CF 2843 on both 2D monolayers and 3D aggregates of A549 lung epithelial cells in agreement with previous studies of biofilms formed *in vitro* in plastic surfaces [39,51]. PelAh, in contrast, did not inhibit biofilm formation in our study, although it has previously been demonstrated to inhibit biofilm formation on plastic surfaces [51]. Our findings, hence, reiterate the therapeutic potential of *Ca*Aly and PslG. We would, however, like to note that we have only examined biofilm inhibition and not eradication of matured/established biofilm in this study.

Whereas animal models are available for cystic fibrosis, the cystic fibrosis mouse models do not replicate the lung environment of the human cystic fibrosis patient, as dominant Clara cells in mouse lungs display phenotypic adaptation and proliferative capacity during injury and inflammation and also express functional CFTR [63,64], mucus clearance and epithelial ion transport in tracheal epithelia are not significantly affected due to the presence of an alternative ion transport channel, Ca^2+^ activated Cl^−^ channel (CaCC), in the cystic fibrosis mouse lung [[65], [66], [67]], and lung pathology differs in cystic fibrosis mice due to differences in size, cellular architecture, host-pathogen interaction, lifestyle, and lifespan [68]. Pig and ferret cystic fibrosis models more closely recapitulate the multi-organ infection of cystic fibrosis [[69], [70], [71]], but are challenging to employ in cystic fibrosis research due to expensive animal husbandry, high neonatal mortality, and slow growth rate [[72], [73], [74]]. Here, we cultured A549 lung epithelial cells as 3D aggregates in a rotary vessel in a low fluid sheer environment using the RCCS system, which is known to be a good model system for studying *in-vivo* host-pathogen interactions and infectious disease mechanisms [75] ^34^. These 3D aggregates are known to mimic the lung environment more closely than monolayers in terms of mucin secretion, presence of tight junctions, cell-cell connections, 3D architecture, and multicellular complexity [33,34,35,37]. The sheer fluid force in the dynamic chamber influences cell proliferation, cell differentiation, and morphology [76,77].

Our demonstration of the efficacy of *Ca*Aly and PslG in inhibiting biofilm formation by cystic fibrosis isolate *P. aeruginosa* CF2843 on the 3D cell aggregates of A549 lung epithelial cells, therefore, highlights the therapeutic potential of these enzymes. Future studies could study the relevance of this strategy for treating *P. aeruginosa* infections in cystic fibrosis by focusing on the efficacy of these enzymes (alone or in combination with suitable antibiotics) in eradicating *P. aeruginosa* biofilms on 3D cell aggregates of cystic fibrosis lung [76] epithelial cells and relevant cystic fibrosis animal models infected with various mucoid and non-mucoid *P. aeruginosa* strains.

## 4 Material and methods

### 4.1 Transposon-mediated insertion of *gfp* in the bacterial chromosome

The previously reported *P. aeruginosa* strain CF2843, a sputum isolate from a cystic fibrosis (CF2843) patient [39] was the strain selected for the chromosomal insertion and expression of GFP. For the GFP insertion, the plasmid pMini-Tn7(Km)-*gfp* and helper plasmid pUXBF13 transformed in appropriate host *E. coli* strains [78] were kindly provided by Professor Soren Molin, Novo Nordisk Foundation Center for Biosustainability, Denmark. These *E. coli* strains were cultured in LB agar at 37 °C with 25 μg/ml kanamycin and 100 μg/ml ampicillin, respectively.

*P. aeruginosa* CF2843 was cultured in LB broth until an OD_600_ of 0.8–1, washed twice with cold water, made electro-competent using electroporation buffer (10% glycerol in water), transformed with helper plasmid pUXBF13 and insertional mini-Tn7-transposon containing plasmid (1 μg each) by electroporation at 2.5 KV, revived in super optimal medium with catabolic repressor (SOC) medium for 3–4 hours at 37 °C on shaking, and then plated on an LB agar plate with 25 μg/ml kanamycin. Mock electroporation (without plasmid) was performed as a control. Screening for *gfp* insertion was by antibiotic selection and by checking for GFP fluorescence upon exposure of the petri plate to UV light.

The stability of the *gfp* insertion in *P. aeruginosa* CF2843GFP was assessed by measuring the fluorescence intensity of the bacterial cell lysates obtained following overnight growth of 5 ml culture broth of *P. aeruginosa strain* CF2843 and *P. aeruginosa strain* CF2843GFP in the presence/absence of kanamycin. Lysis was performed with the help of bacterial cell lysis buffer (10 mM Tris, 100 mM NaCl, 1 mM EDTA, and 1 mg/ml Lysozyme at pH 8.0) for 2 hours at 37 °C. The lysed culture was clarified by high-speed centrifugation, and the fluorescence intensity was measured (λ_ex_: 480 nm and λ_em_: 510 nm) using a Synergy Hybrid plate reader H1. The procedure was repeated for five consecutive generations.

### 4.2 Whole genome analysis

Genomic DNA was isolated from a 5 ml culture of *P. aeruginosa* using QuickDNA Fungal/Bacterial miniprep kit (Zymo D6005), and its integrity was evaluated on a 1% agarose gel (Lonza, Belgium). DNA purity and concentration were assessed using a Nanodrop 2000 (Thermofisher Scientific, Massachusetts, USA). The DNA was further purified using AMPure PB beads (Pacific Biosciences), and the final concentration was measured with a Qubit 4.0 fluorometer with the DNA HS assay kit (Thermofisher Scientific, Massachusetts, USA). The size distribution of the genomic DNA was determined using an Agilent FEMTO Pulse analyzer (Agilent, California, USA). SMRTbell libraries were prepared with the Express Template Prep Kit 2.0 (Pacific Biosciences). Before library preparation, the genomic DNA was sheared to a target size of 7–10 kb using a Megaruptor 3 (Diagenode, Belgium). Single-strand overhangs created during shearing were removed, followed by DNA damage repair and end-repair/A-tailing. SMRTbell adapters were ligated to the end-repaired DNA fragments, and hairpin dimers formed during the ligation process were removed using AMPure PB beads. The final adapter-ligated SMRTbell libraries were evaluated using the Agilent FEMTO Pulse analyzer. Primer annealing and polymerase binding to the pooled and purified SMRTbell library were done to prepare the bound complex using the Sequel II binding kit 2.2 (Pacific Biosciences). Approximately 90 pM of the library was loaded onto one SMRTcell containing 8 M zero-mode waveguides (ZMWs) and sequenced on a PacBio Sequel II system in circular consensus sequencing (CCS)/HiFi mode (Nucleome Informatics Pvt. Ltd.). The ccs tool (v6.4.0) was used to generate circular consensus sequences from the subreads and resulted in 73,873 reads with 750,511,348 bp [79]. The reads were then filtered for quality using Filtlong v0.2.1 (https://github.com/rrwick/Filtlong), discarding the worst 5% of reads and those shorter than 1 kbp, resulting in 70,634 reads (712,995,345 bp) with an average read length of 11,322 bp. A de novo assembly was generated using Trycycler v0.5.4 with default parameters [80]. The final genome size was 6,350,848 bp with a GC content of 66.46% and coverage of 112X. Gene prediction and functional annotation were performed using Bakta v1.5.1 ^81^, revealing a coding density of 90.3% and 5792 coding sequences (CDS).

#### 4.2.1 Phylogenetic analysis

We retrieved a total of 144 complete genome sequences of *P. aeruginosa* (73 from non-cystic fibrosis individuals and 71 from cystic fibrosis patients, as per the metadata associated with the submitted entries) from the *Pseudomonas* Genome DB (https://www.pseudomonas.com/) [82]. We identified their sequence types by scanning the sequences against the PubMLST database [83] using the mlst tool (accessed on June 25, 2024) (https://github.com/tseemann/mlst) [84]. A total of six housekeeping genes (*acsA*, *aroE*, *guaA, mutL, nuoD*, and *ppsA*) of *P. aeruginosa* were retrieved from all the selected 146 genomes (including our sequenced strain CF2843GFP, an outgroup *P. aeruginosa* PA7, and the aforementioned 144 genomes). Further, the sequences were aligned and concatenated using the PhyloSuite tool (v1.2.3) [85] to construct a maximum likelihood (ML) tree using discrete Gamma distribution at Invariable sites (G + I) of the T92 substitution model included in MEGA (v11.0.13) [86] with a bootstrap of 100. Another SNP (single nucleotide polymorphisms) based phylogenetic tree was built by considering all 146 complete genomes, with *P. aeruginosa* PA7 as the outgroup, using Parsnp (v2.0.5) [87]. Both the mlst and SNP-based phylogenetic trees were visualized using iTOL (v6.9.1) [88].

#### 4.2.2 Identification and analysis of functional genes

We identified all genes across the 144 selected *P. aeruginosa* genomes using Bakta (v1.3.3) [81], and looked for the functional genes associated with biofilm formation (*psl* and *pel*), alginate biosynthesis, and antimicrobial resistance (AMR). Further, the BLASTP search (identity ≥90% and e-value < 1e-5) was performed against biofilm formation and alginate biosynthesis genes in *P. aeruginosa* PAO1 present in *Pseudomonas* Genome DB. The AMRFinderPlus (v3.12.8) [89], ARGfams (v0.5.0) [90], and RGI 6.0.3 tool in CARD 3.2.9 [https://card.mcmaster.ca/analyze/rgi] were used to identify the AMR genes across all the selected 144 *P. aeruginosa* genomes. For data accuracy, only genes predicted by all three tools were considered AMR genes in selected strains. A DIAMOND BLASTP search (identity ≥90% and e-value < 1e-5) was performed against 4236 core datasets of virulent genes present in the VFDB database (as of June 26, 2024) to identify the virulent genes in our isolated strain CF2843GFP. The circular genome plot of strain *P. aeruginosa* CF2843GFP with selected functional genes was visualized using Proksee [91].

### 4.3 Expression and purification of *Ca*Aly, PslG, and PelAh

Recombinant *Ca*Aly and PslG were expressed and purified, as reported previously [39]. The PelAh gene was custom synthesized (GenetoProtein Pvt Ltd) in vector pET-28a(+) with a 6XHis tag. The recombinant protein PelAh was successfully overexpressed in *E. coli* BL21 (DE3) cells by inducing expression with 0.1 mM IPTG (GoldBio) and incubating at 22 °C for 16 hours with shaking at 160 rpm. The bacterial cells were then pelleted down at 4000*×g* for 7 min at room temperature. The pellet was resuspended in lysis buffer (20 mM Tris, 150 mM NaCl, pH 7.5) and disrupted by Probe-type ultrasonicator (Sonics and Materials NC) for 30 min at 30% amplitude with 10 s off 10 s on pulse cycles, followed by centrifugation at 16000*×g* for 40 min. The clarified lysate was allowed to bind to Ni-NTA resin with end-over-end rotation at 4 °C for 2 hours. The resin was then extensively washed with Tris-buffered saline (TBS; 20 mM Tris, 150 mM NaCl, pH 7.5) containing 30 mM imidazole (Sigma) and eluted with TBS containing 250 mM imidazole. The recombinant protein was then extensively dialyzed against TBS, and the purity and yield were assessed by SDS-PAGE and OD_280_, respectively. The recombinant proteins, *Ca*Aly and PslG, were purified using metal ion affinity chromatography as previously described [39].

### 4.4 Transmission electron microscopy (TEM)

Overnight cultures of the clinical strain of *P. aeruginosa* CF2843 and *P. aeruginosa* CF2843GFP were washed twice with sterile water and diluted to 10^6^ cfu/ml. The TEM sample was prepared by placing one or two drops in a pure carbon 300 mesh Cu grid (Ted pella, 01843-F) under ambient conditions. The sample on the grid was then scanned using Jeol Jem 2100 Transmission electron microscope (In-house Electron Microscopy facility).

### 4.5 Culture of 3D aggregates of A549 cells using rotary cell culture system (RCCS)

The A549 lung epithelial cells were first grown in the complete growth medium, which contained Ham's F–12K (Kaighn's) medium supplemented with 10% Fetal Bovine Serum and 1% penicillin-streptomycin, as monolayer culture up to 90% confluency. After reaching 90% confluence, cells were detached using 2 ml of 0.1% Trypsin-EDTA and harvested by centrifugation. A hemocytometer was used to count the number of viable cells following trypan blue staining.

A Slow-Turning Lateral Vessel (STLV) of the Rotary Cell Culture System (Synthecon) of 55 ml volume was used to generate 3D cell aggregates of lung epithelial cells using Cytodex-3 collagen-coated microcarrier beads (Sigma C3275). First, Cytodex-3 microcarrier beads (50 mg) were soaked in PBS for 3–4 hours, washed twice with PBS, autoclaved at 121 °C for 20 min, stored at 4 °C, and brought to room temperature before use. Then, 5X10^6^ cells/ml were incubated with the Cytodex-3 microcarrier beads for 20 min at room temperature and a further 20 min in an incubator at 37 °C, 5% CO_2_. The vessel was then filled with the complete growth medium, inoculated with the mixture of beads and cells, attached to the rotor base, and kept incubated at 37 °C, 5% CO_2_, with a rotor speed of 9–14. The vessel medium was changed, and the culture was monitored daily. All further experiments were performed on days 9–16 of the 3D cell culture.

### 4.6 Scanning electron microscopy (SEM)

A silicon wafer (SI WFR, cat no16006, Ted Pella Inc.) was freshly coated with 1 mg/ml of poly-l-lysine for 2 hours at room temperature. Overnight LB cultures of *P. aeruginosa* CF2843 and *P. aeruginosa* CF2843GFP were incubated on the poly-l-lysine coated silicon wafer for 6 hours to allow for biofilm formation [92]. For experiments with 3D cell aggregates, 3D cell aggregates were allowed to attach to the wafer overnight, and *P. aeruginosa* CF2843GFP was then allowed to form biofilms for 6 hours on the 3D aggregates. The samples were then fixed with 4% glutaraldehyde for 4 hours at room temperature, rinsed twice with PBS and twice with sterile water, and dehydrated with graded ethanol, 25%, 50%, 75%, and 90% for 10 min each, and then twice with 100% ethanol for 15 min each [93]. The wafer was air-dried overnight in a laminar flow hood at room temperature, and then proceeded with gold spur coating to reduce the electric charge and to maintain the homogenous sample surface. The samples were then subjected to Field Emission – Scanning Electron Microscopy (FE-SEM) using a Jeol FE-SEM at Thapar Institute of Technology & Technology, Punjab.

### 4.7 Immunofluorescence and confocal microscopy

#### 4.7.1 Phalloidin staining in 3D aggregates of lung epithelial cells

The cultured 3D aggregates of A549 cells were harvested on day 10 and allowed to attach overnight to the four-chamber imaging slide (Cell Imaging Coverglass, Eppendorf, cat. no. 0030742028). The culture media was then removed, and the 3D cell aggregates were washed once with PBS. The 3D aggregates were fixed using 4% paraformaldehyde for 20 min at room temperature, washed twice with PBS, permeabilized with 0.1% Triton-X-100 in PBS for 5 min, and again washed twice with PBS. The 3D aggregates were then incubated with 0.5 μl of Alexa Flour 568 Phalloidin (Thermo Scientific, cat. no. A12380) in 100 μl PBS for 1 hour at room temperature to stain F-actin filaments, washed twice with PBS, and incubated with a 1:5000 dilution of 1 mg/ml DAPI (Thermo Scientific, cat. no. 62248) for 15 min at room temperature to stain the nucleus. Confocal microscopy (Nikon-A1(R)) was performed using lasers of 568 nm and 405 nm, respectively (in-house confocal microscopy facility).

#### 4.7.2 Immunocytochemistry of monolayers and 3D aggregates of lung epithelial cells

The A549 monolayers and 3D aggregates were assayed for cell surface fucosylation and sialylation with the help of 10 μg/ml biotinylated l-fucose-specific *Aleuria aurantia* lectin [94], α-2,3 sialic acid-specific *Maackia amurensis* lectin (MAL-II), and α-2,6 sialic acid-specific *Sambucus nigra* agglutinin (SNA) (all from Vector Labs). Streptavidin-conjugated Alexa Fluor 555 (AF555) was used at a dilution of 1:1000 to detect lectin binding, and confocal microscopy was performed using a laser of 568 nm (in-house confocal microscopy facility).

Cell surface mucin was detected using mouse anti-Muc1 (Genetex, cat. no. GTX100459) and mouse anti-Mucin 5Ac (Genetex, cat. no. GTX11335), both used at a dilution of 1:100. The Alexa Fluor 488 (AF488)-tagged donkey anti-mouse IgG (Jackson Laboratories) antibody at a dilution of 1:800 was employed as the secondary antibody, and confocal microscopy was performed using a laser of 488 nm (in-house confocal microscopy facility).

Tight junctions were detected with rabbit anti-beta-catenin (Genetex, cat. no. GTX101435) at a dilution of 1:50, followed by the secondary antibody AF488-tagged goat anti-rabbit antibody (Thermo Scientific, cat. no. A11088) at a concentration of 5 μg/ml, and confocal microscopy was performed using a laser of 488 nm (in-house confocal microscopy facility).

The immunocytochemistry procedure was performed as follows. Three-dimensional aggregates of lung epithelial A549 cells were harvested from the RCCS system and placed in a chamber glass slide (Eppendorf, cat. no. 0030742036), fixed with 4% paraformaldehyde (PFA; Thermo Scientific, cat. no. 28908), and then washed twice with PBS. Similarly, a monolayer of lung epithelial cells was grown on a poly-l-lysine coated coverslip placed on a six-well plate by allowing cells to adhere and form a confluent monolayer for 24 hours, fixed with 4% PFA, and washed twice with PBS. The primary antibody or biotinylated lectin in PBS containing 1% BSA was added to the slide for 3 hours at room temperature, and the slides were washed thrice with PBS. The fluorophore-conjugated secondary antibody or streptavidin in PBS containing 1% BSA was added for 45 min at room temperature, and the slides were washed thrice with PBS. SlowFade Diamond anti-fade (Thermo, S36963) was used to mount the cells, and the fluorescence was measured using confocal microscopy with the appropriate laser as mentioned above.

### 4.8 Biofilm formation and inhibition

For confocal imaging of biofilm formation, *P. aeruginosa* CF2843GFP was cultured in LB broth medium for 6 hours and diluted to 1X10^7^ cells/100 μl. The 3D aggregates and the monolayers of lung epithelial cells were infected with clinical *P. aeruginosa* CF2843GFP at an MOI of 20:1 for 6 hours at 37 °C, 5% CO_2_. For biofilm inhibition, commercially available antibiotic colistin (at final concentrations of 0.1 μg/ml, 1 μg/ml, and 10 μg/ml), 5 μM alginate lyase *Ca*Aly, 5 μM Psl hydrolase PslG, and 5 μM Pel hydrolase PelAh (from 10X stocks of 50 μM) were added after 1 hour of infection; TBS was used for a negative control. After 6 hours of infection, slides were washed twice with PBS and then sealed and subjected to confocal microscopy with a laser of 488 nm wavelength.

For quantitating the inhibition of biofilm formation, 1X10^4^ A549 lung epithelial cells (3D aggregates or monolayer) were seeded in a 96-well plate and incubated overnight/until confluency (for 3D aggregates/monolayers, respectively). Cells were infected with *P. aeruginosa* CF2843 at an MOI of 20:1 for 12 hours. After an hour of infection, colistin (final concentrations of 0.1 μg/ml, 1 μg/ml, and 10 μg/ml) or 10 μl of 50 μM *Ca*Aly, PslG, or PelAh were added into the well plate. After 12 hours of treatment, the supernatant was removed, and the wells were washed with PBS to remove the planktonic cells. The cells were treated with 100 μl of 0.1% Triton-X-100 for 30 min at 37 °C to remove the attached cells and then serially diluted in LB broth, and 100 μl of 10^−5^, 10^−6^, and 10^−7^ dilutions were plated on LB agar plate and incubated at 37 °C for 16–18 hours. The number of colonies was counted and noted. The experiment was performed thrice, each time in duplication (three different cultures of *P. aeruginosa* and three different preparations of *Ca*Aly, PslG, and PelAh).

## CRediT authorship contribution statement

**Neetu:** Writing – review & editing, Writing – original draft, Validation, Methodology, Investigation, Formal analysis. **Shilpee Pal:** Writing – review & editing, Methodology, Investigation, Formal analysis. **Srikrishna Subramanian:** Writing – review & editing, Supervision, Software, Methodology, Investigation, Formal analysis. **T.N.C. Ramya:** Writing – review & editing, Supervision, Project administration, Methodology, Funding acquisition, Formal analysis, Conceptualization.

## Funding

This work was supported by the 10.13039/501100001412Council of Scientific and Industrial Research, Government of India (CSIR-IMTECH Research Council-approved project OLP0554 to T.N.C. Ramya). Neetu acknowledges the University Grants Commission, Government of India, for her fellowship. Shilpee Pal acknowledges the Indian Council of Medical Research (ICMR) for financial assistance under the ICMR-Research Associateship (File Number- BMI/11(48)/2022; 2021–11207).

## Declaration of competing interest

The authors declare that they have no known competing financial interests or personal relationships that could have appeared to influence the work reported in this paper.

## Data Availability

Data will be made available on request.
