# Supplementary material for: Cellulophaga algicola alginate lyase and Pseudomonas aeruginosa Psl glycoside hydrolase inhibit biofilm formation by Pseudomonas aeruginosa CF2843 on three-dimensional aggregates of lung epithelial cells
Source: Biofilm. 2025 Feb 22;9:100265. doi: 10.1016/j.bioflm.2025.100265 (PMC11891150; doi:10.1016/j.bioflm.2025.100265)
Supplement: Multimedia component 1 [file mmc1.docx]

**SUPPLEMENTARY FIGURES S1-S5**

***Cellulophaga algicola* alginate lyase and *Pseudomonas aeruginosa* Psl glycoside hydrolase inhibit biofilm formation by *Pseudomonas aeruginosa* CF2843 on three-dimensional aggregates of lung epithelial cells**

**Supplementary Figure 1**

**
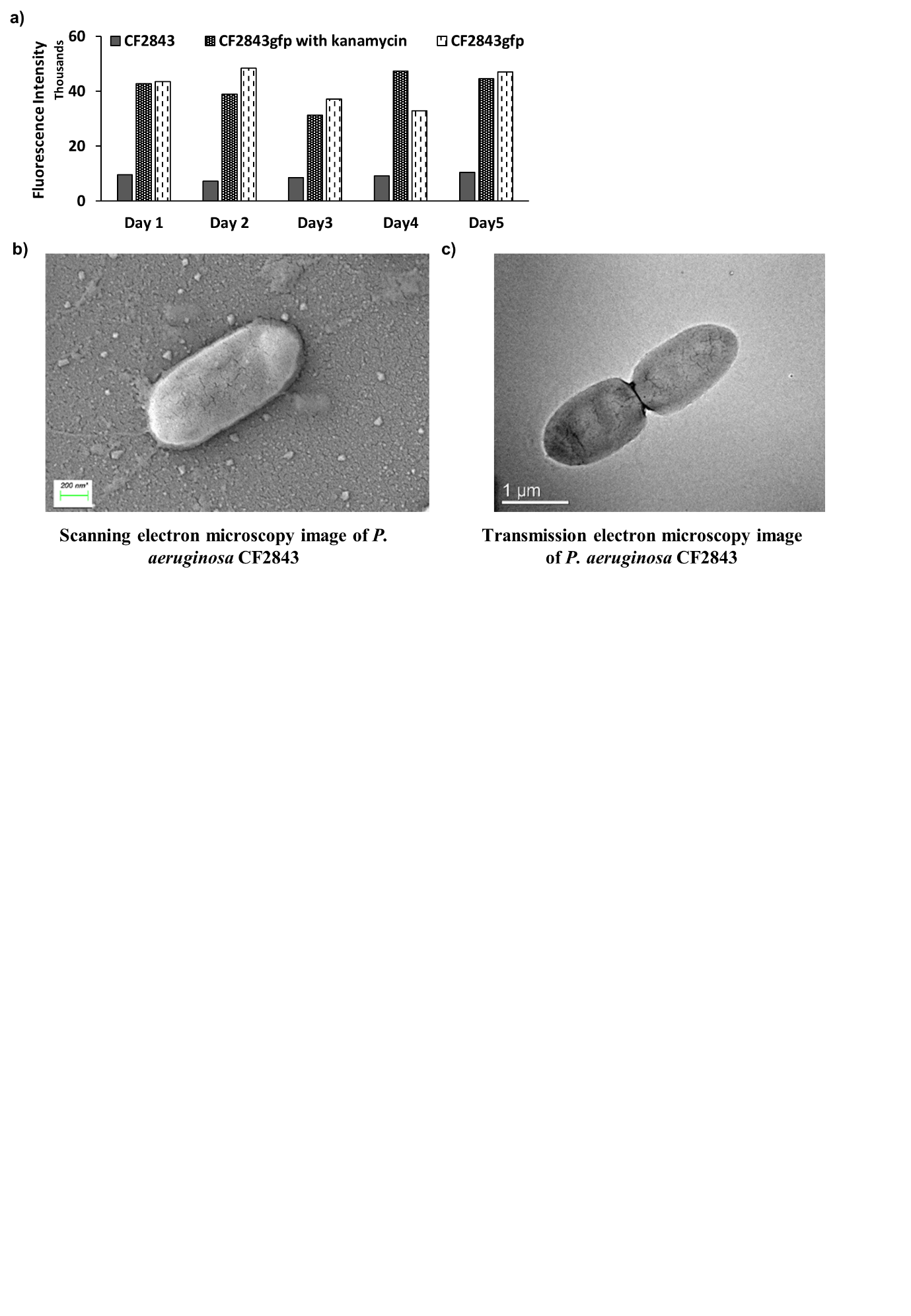
Supplementary Figure 1: Stability of *gfp* integration in *P. aeruginosa* CF2843 and cellular morphology of *P. aeruginosa* CF2843. a)** GFP fluorescence indicating the stable integration of *gfp* in *P. aeruginosa* CF2843GFP over five consecutive subculturing periods. **b)** Scanning Electron Microscopy image of *P. aeruginosa* CF2843 (scale bar: 200 nm). **c)** Transmission Electron Microscopy image of *P. aeruginosa* CF2843 (scale bar: 1 µm).

**Supplementary Figure 2**

**
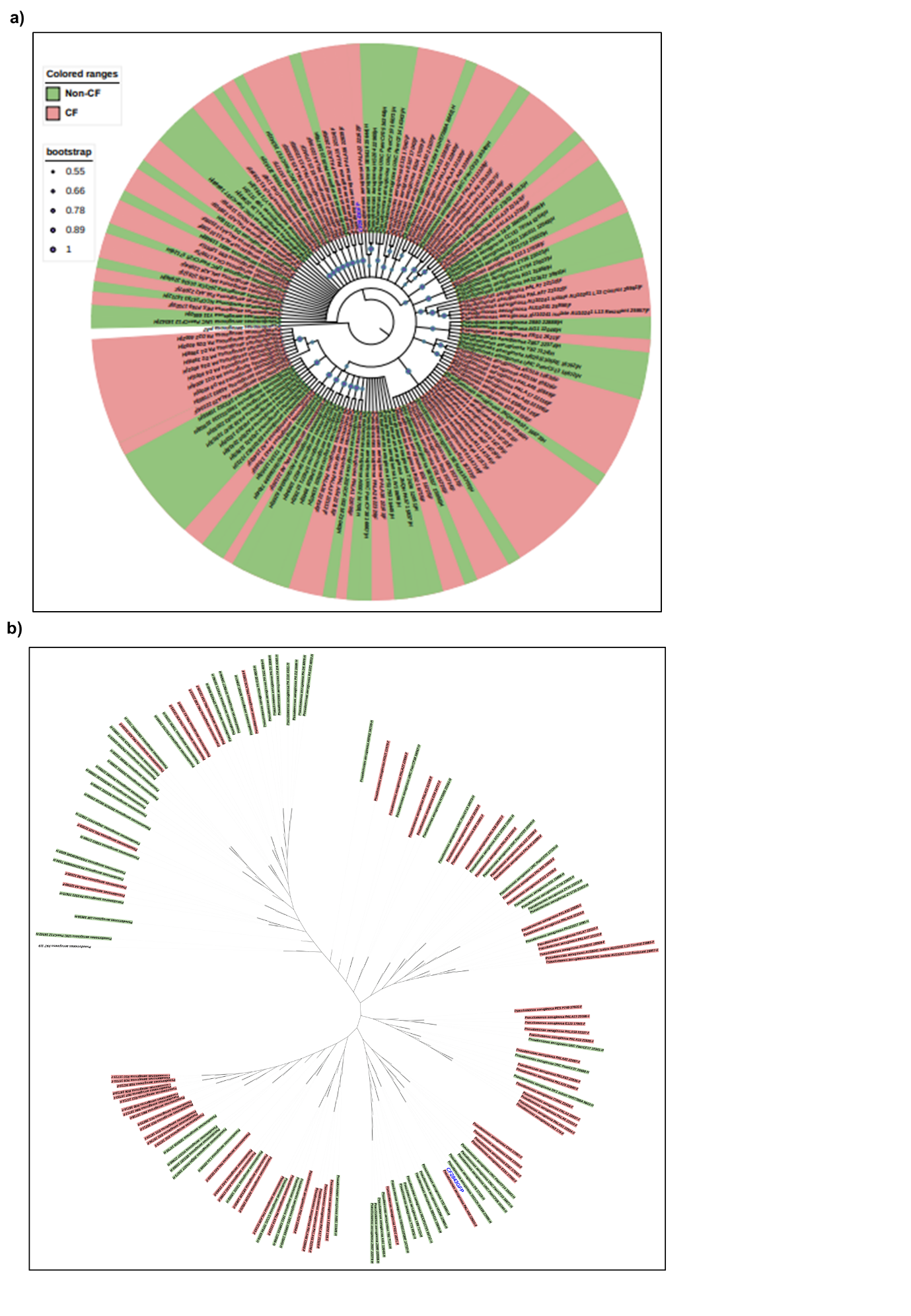
Supplementary Figure 2: Phylogenetic positioning of *P. aeruginosa* CF2843. a)** MLST-based phylogenetic tree of *P. aeruginosa* CF2843GFP and *P. aeruginosa* isolates from cystic fibrosis (red) and non-cystic fibrosis individuals (green). *P. aeruginosa* PA7 was used as the outgroup. **b)** SNP-based phylogenetic tree of *P. aeruginosa* CF2843GFP and *P. aeruginosa* isolates from cystic fibrosis (red) and non-cystic fibrosis individuals (green). *P. aeruginosa* PA7 was used as the outgroup.

**Supplementary Figure 3**

**
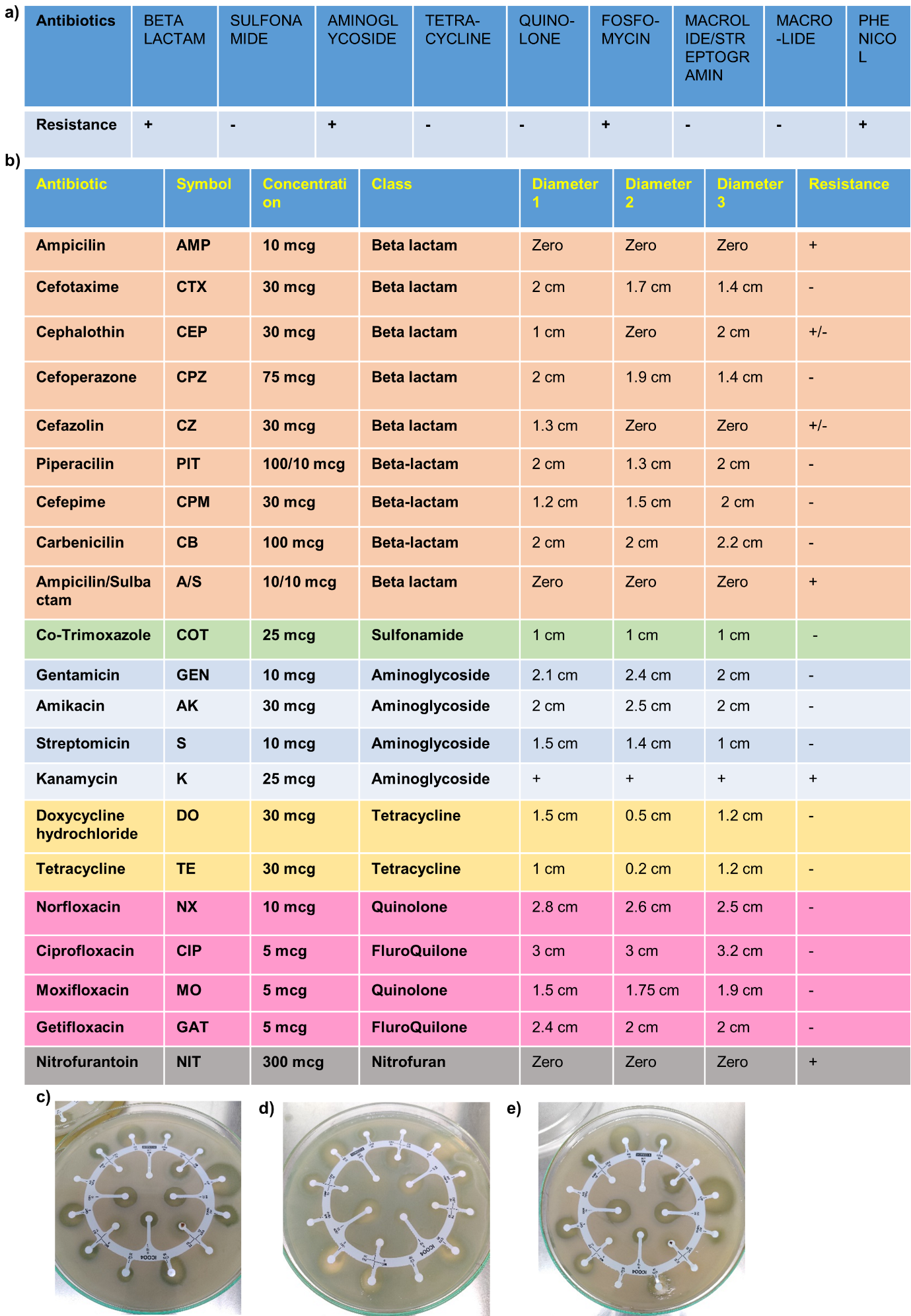
**

**Supplementary Figure 3:** AMR in *P. aeruginosa* CF2843GFP*.* **a)** AMR genes predicted to be present in *P. aeruginosa* CF2843GFP*.* **b)** Antibiotic resistance profile of *P. aeruginosa* CF2843GFP*.* **c-d)** Agar plates indicating the antibiotic resistance profile of *P. aeruginosa* CF2843GFP*.*

**Supplementary Figure 4**

**
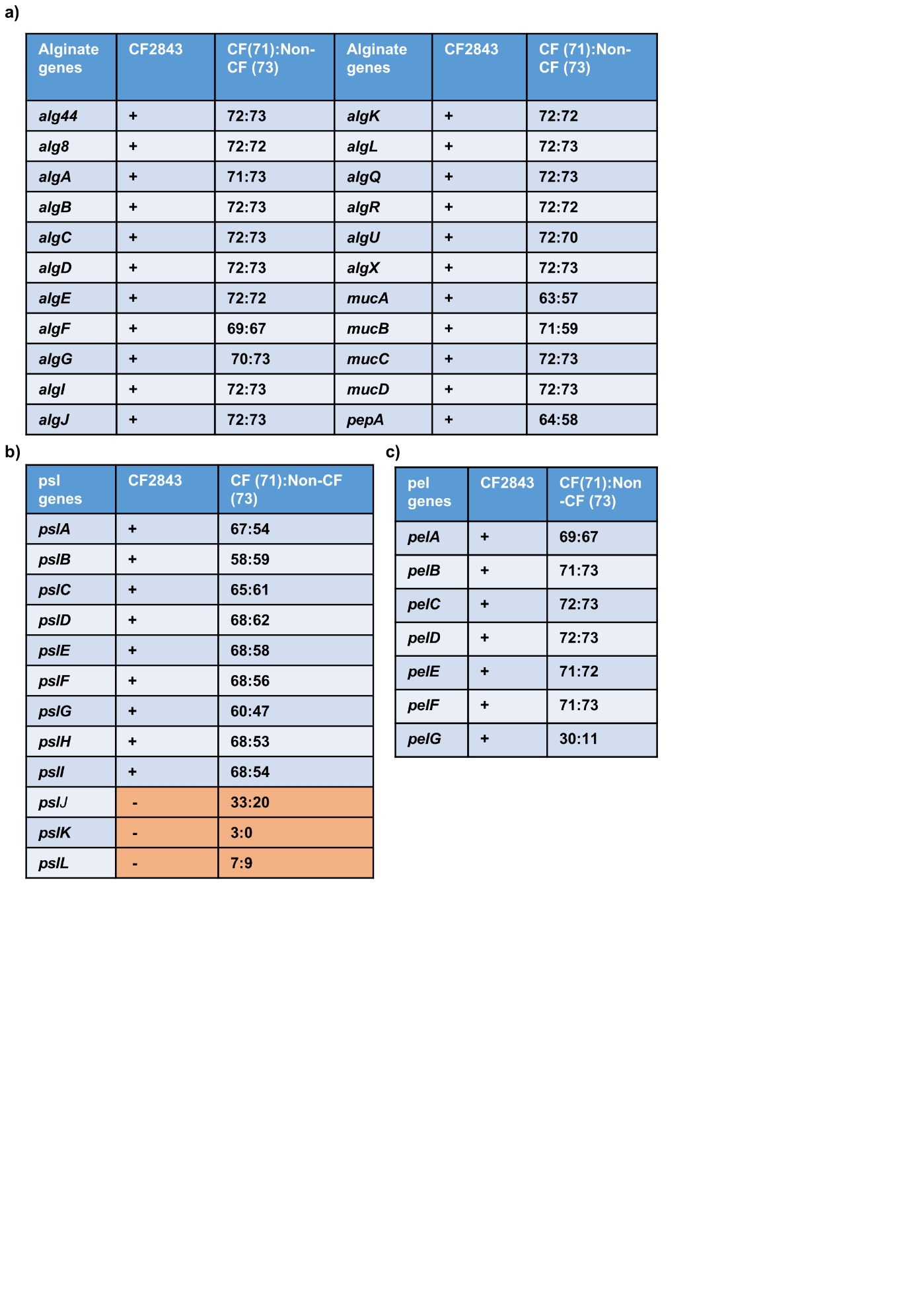
**

**Supplementary Figure 4:** Exopolysaccharide biosynthesis in *P. aeruginosa.* **a)** Alginate biosynthesis pathway genes predicted to be present in *P. aeruginosa* CF2843GFP and different isolates of *P. aeruginosa* from cystic fibrosis (CF) and non-cystic fibrosis (non-CF) individuals. **b)** Psl biosynthesis pathway genes predicted to be present in *P. aeruginosa* CF2843GFP and different isolates of *P. aeruginosa* from cystic fibrosis (CF) and non-cystic fibrosis (non-CF) individuals. **c)** Pel biosynthesis pathway genes predicted to be present in *P. aeruginosa* CF2843GFP and different isolates of *P. aeruginosa* from cystic fibrosis (CF) and non-cystic fibrosis (non-CF) individuals.

**Supplementary Figure 5**

**
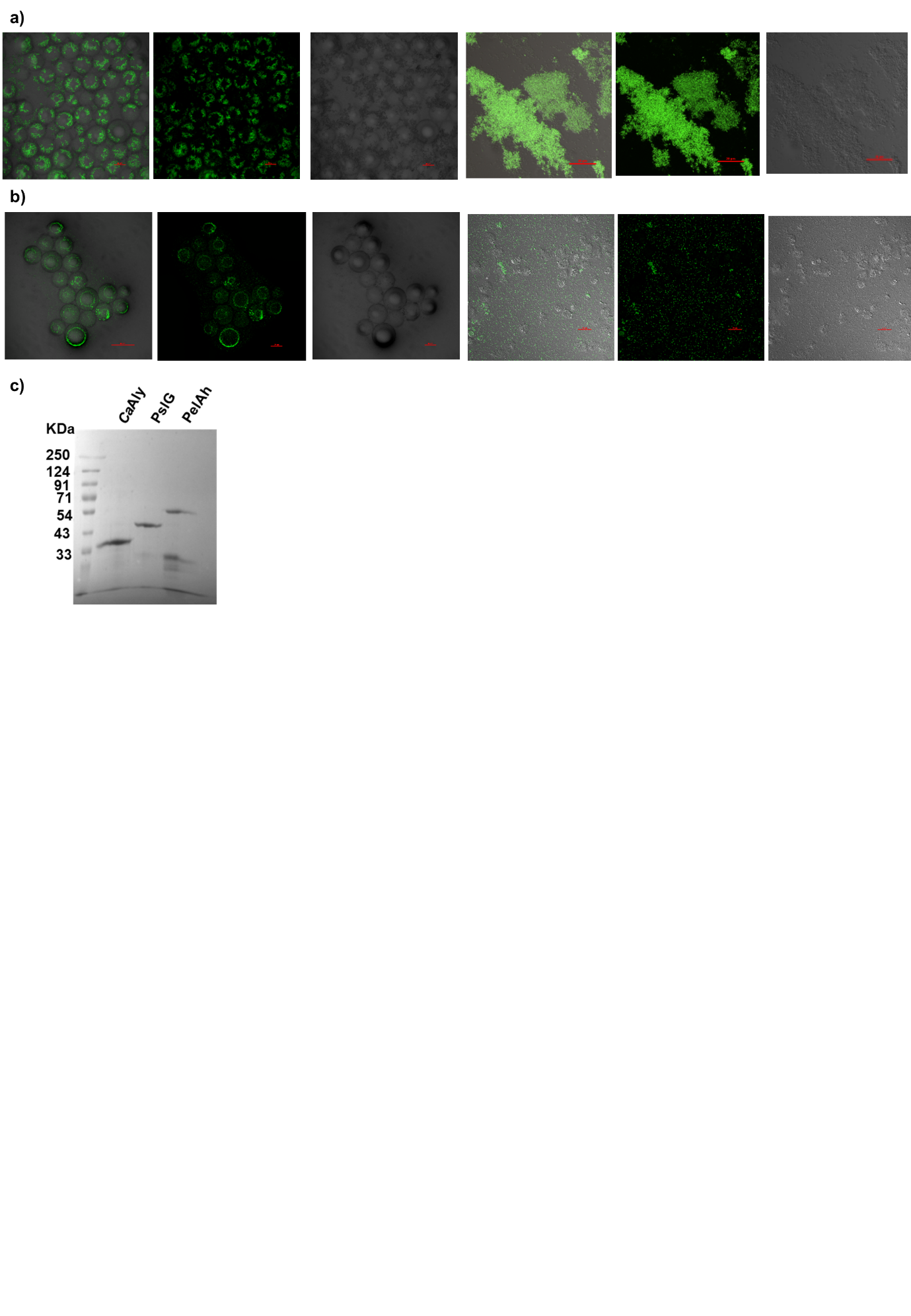
**

**Supplementary Figure 5:** **Biofilm formation *by P. aeruginosa* CF2843GFP on A549 lung epithelial cells and its inhibition. a)** Confocal microscopy image showing *P. aeruginosa* CF2843GFP biofilms on 3D aggregates and monolayers of A549 cells after 12 hours of incubation with 0.1 µg/ml colistin (Scale bars for 3D aggregates and monolayers are 200 µm and 20 µm, respectively). **b)** Confocal microscopy image showing *P. aeruginosa* CF2843GFP biofilms on 3D aggregates and monolayers of A549 cells after 12 hours of incubation with 1 µg/ml colistin (Scale bars for 3D aggregates and monolayers are 100 µm and 20 µm, respectively). **c)** Image of Coomassie blue stained polyacrylamide gel following SDS-PAGE showing the purified preparations of *Ca*Aly, PslG, and PelAh used in the biofilm inhibition experiments.
